# A toolbox for systematic discovery of stable and transient protein interactors in baker's yeast

**DOI:** 10.15252/msb.202211084

**Published:** 2023-01-18

**Authors:** Emma J Fenech, Nir Cohen, Meital Kupervaser, Zohar Gazi, Maya Schuldiner

**Affiliations:** ^1^ Department of Molecular Genetics Weizmann Institute of Science Rehovot Israel; ^2^ The de Botton Protein Profiling Institute of the Nancy and Stephen Grand Israel National Centre for Personalized Medicine Weizmann Institute of Science Rehovot Israel

**Keywords:** interaction profiling, substrate discovery, TurboID, yeast libraries, Methods & Resources, Post-translational Modifications & Proteolysis, Proteomics

## Abstract

Identification of both stable and transient interactions is essential for understanding protein function and regulation. While assessing stable interactions is more straightforward, capturing transient ones is challenging. In recent years, sophisticated tools have emerged to improve transient interactor discovery, with many harnessing the power of evolved biotin ligases for proximity labelling. However, biotinylation‐based methods have lagged behind in the model eukaryote, *Saccharomyces cerevisiae*, possibly due to the presence of several abundant, endogenously biotinylated proteins. In this study, we optimised robust biotin‐ligation methodologies in yeast and increased their sensitivity by creating a bespoke technique for downregulating endogenous biotinylation, which we term ABOLISH (Auxin‐induced BiOtin LIgase diminiSHing). We used the endoplasmic reticulum insertase complex (EMC) to demonstrate our approaches and uncover new substrates. To make these tools available for systematic probing of both stable and transient interactions, we generated five full‐genome collections of strains in which every yeast protein is tagged with each of the tested biotinylation machineries, some on the background of the ABOLISH system. This comprehensive toolkit enables functional interactomics of the entire yeast proteome.

## Graphical abstract

This study presents a novel method for enhancing biotin‐specific signal relative to noise and a complete protein interaction toolbox, which harnesses the power of exogenous biotin ligases in discovering stable and transient protein interactors in yeast.

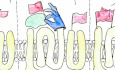

## Introduction

Cellular architecture and function require the action of protein machines often formed by protein complexes. Characterising the subunit identity of such complexes can be done using classic protein–protein interaction (PPI) assays such as immunoprecipitation (IP) of an epitope‐tagged protein followed by mass spectrometry (MS) (Dunham *et al*, 2012). However, to uncover their protein substrates and their regulators (such as posttranslational modification enzymes), it is essential to probe transient interactions. Transient PPIs cannot easily be captured by simple IP approaches since the nature of these experiments (such as the lysis conditions and long incubation times) selects only stable interactions between machinery subunits and cofactors. Therefore, when searching for transient PPIs between machineries and their clients or regulators, a more specialised approach is required.

One such approach is that of proximity labelling (PL) in which proteins proximal to an active enzyme are marked by a covalent tag that can be identified long after the interaction has ended. Biotin ligation represents a central PL method; with the first approach developed from the endogenous *Escherichia coli* biotin ligase, BirA (Cronan, 1990). BirA specifically biotinylates a lysine (K) residue within a short acceptor peptide sequence (Avi) (Beckett *et al*, 1999) in the presence of free biotin and ATP. Therefore, by tagging one protein with BirA and another with an Avi sequence (termed AviTag), stable and transient PPIs can be assessed in a pairwise manner using the high‐affinity biotin binder, streptavidin, to detect biotinylated AviTag.

Having a pairwise assay enabled hypothesis‐driven experiments but was less amenable to unbiased interactor discovery. Hence, a huge leap in the ability to utilise BirA‐based methods for *de novo* discovery of interactions came with the creation of a promiscuous BirA mutant, BirA_R118G_ (Choi‐Rhee *et al*, 2004). This mutant is able to biotinylate available K residues on accessible proteins without the requirement for a specialised acceptor sequence, making it possible to capture and identify multiple biotinylated interactors in one experiment using streptavidin affinity‐purification (AP)‐MS. Indeed, this powerful tool, named BioID, was shown to enable the discovery of new PPIs in mammalian cells (Roux *et al*, 2012). Later, a smaller version of BioID (BioID2) was generated from the *Aquifex aeolicus* BirA (Kim *et al*, 2016). However, the most active biotin ligase to date, TurboID, was produced by the directed evolution of a BioID variant in *Saccharomyces cerevisiae* (from here on termed simply yeast) (Branon *et al*, 2018).

Surprisingly, despite the use of yeast to evolve TurboID, there has been limited use of these systems in yeast. BioID has so far principally been applied to elucidate PPIs for ribosome‐ and mitoribosome‐associated proteins (Opitz *et al*, 2017; Singh *et al*, 2020). One reason for this may be that BioID functions optimally at 37°C (Kim *et al*, 2016) and is minimally active at 30°C—the temperature at which yeast is normally cultured. TurboID, on the contrary, displays high activity at 30°C (Branon *et al*, 2018) and was employed to discover interactors for soluble cytosolic and exosomal proteins in the fission yeast, *Schizosaccharomyces pombe* (Larochelle *et al*, 2019), but remains untested for PPI discovery in *S. cerevisiae*. A more general reason explaining why biotin‐based approaches have lagged behind in this powerful model organism is the presence of several highly expressed native proteins that are endogenously biotinylated (Sumrada & Cooper, 1982; Stucka *et al*, 1991; Hasslacher *et al*, 1993; Brewster *et al*, 1994; Hoja *et al*, 2004; Kim *et al*, 2004; Nagaraj *et al*, 2012). These proteins hence make up a significant proportion of the signal following enrichment of biotinylated proteins and can therefore reduce the chance of identifying interactors—especially if they are low‐abundance proteins or transient in nature.

Clearly, biotinylation‐based tools have immense power to uncover PPIs as has been demonstrated in multiple model systems, including mammalian cells (Roux *et al*, 2012; Go *et al*, 2021), mice (Uezu *et al*, 2016; Kim *et al*, 2021; Liu *et al*, 2021), flies (Uçkun *et al*, 2021), worms (Artan *et al*, 2021; Sanchez *et al*, 2021) and plants (Mair *et al*, 2019; Zhang *et al*, 2019). This widespread utilisation incentivised our work to make this tool applicable for the systematic identification of PPIs, particularly transient ones, in yeast. To this end, we address the current gap in PL technology in yeast by optimising protocols for the discovery of stable and transient interactions using a variety of biotin‐ligation‐dependent techniques. Moreover, we developed a novel approach, which we term ABOLISH (Auxin‐induced BiOtin LIgase diminiSHing), for the downregulation of endogenous biotinylation to increase the signal‐to‐noise ratio and make PPI discovery more robust. We showcase the power of these approaches by uncovering a set of new substrates for the endoplasmic reticulum (ER) localised insertase, the ER membrane complex (EMC). Most importantly, to enable these powerful tools to be used easily and rapidly by the entire yeast community and to promote systematic probing of interactions, we generated a collection of full‐genome libraries in which each yeast gene is preceded by either TurboID‐HA, BioID2‐HA, BirA, or AviTag, with the ABOLISH system integrated into several of them. Altogether these freely available libraries provide a powerful platform for high‐content PPI screening and ultimately substrate recognition and protein function discovery in yeast.

## Results

### Developing ABOLISH—a strategy to enhance the signal‐to‐noise ratio for exogenous biotin ligases

**Figure 1:**
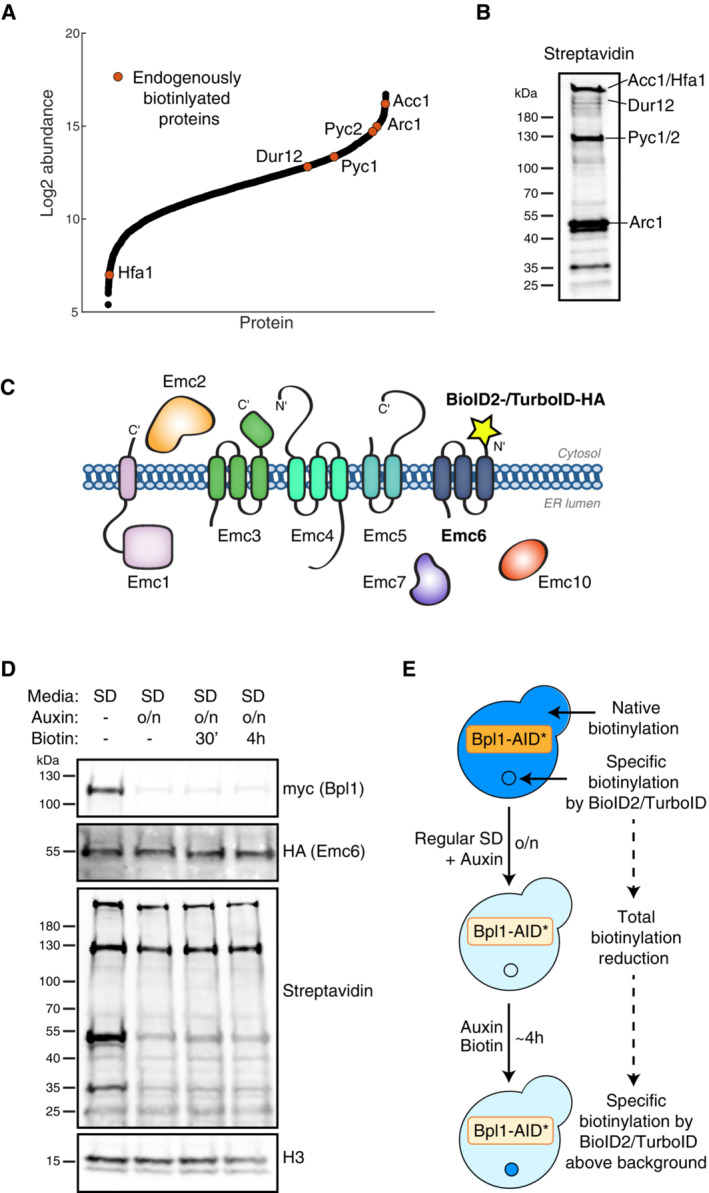
Utilising an auxin‐inducible degron to reduce levels of endogenous biotinylation in TurboID‐expressing cells The abundance of endogenously biotinylated proteins (orange circles) relative to the abundance of all yeast proteins (black circles) as determined by Nagaraj *et al* (2012).A streptavidin blot of a control strain showing the running pattern of the endogenously biotinylated proteins highlighted in (A) and labelled according to Pirner and Stolz (2006).Schematic of the EMC showing Emc6 N‐terminally tagged with BioID2‐HA or TurboID‐HA.Western blot analysis of a strain expressing TurboID‐HA‐Emc6, Bpl1‐AID*‐9myc and OsTIR1, which was grown overnight in SD media with or without auxin. The cells were then diluted into fresh SD media and grown to mid‐logarithmic phase (~ 4 h) with or without auxin, respectively. When required, biotin was added either 30 min before harvesting, or for the entire 4 h. H3 (histone H3) is used as a loading control.Schematic of the growth conditions used in (D), which were selected for the remaining ABOLISH experiments.

To expand the arsenal of biotin‐based tools available for protein interaction profiling in yeast it is essential to take into consideration endogenously biotinylated proteins (Sumrada & Cooper, 1982; Stucka *et al*, 1991; Hasslacher *et al*, 1993; Brewster *et al*, 1994; Hoja *et al*, 2004; Kim *et al*, 2004). This is because most biotinylated yeast proteins are highly abundant (Fig 1A) and therefore can mask many of the expected PPI assay signals on a streptavidin blot (Fig 1B) or take up a large percent of the reads from MS analyses. Since PL relies on biotinylated protein enrichment and detection, it would clearly be advantageous to reduce the background signal from the endogenously biotinylated proteins. To do this, we created a new method of endogenous biotinylation reduction that we call ABOLISH, for Auxin‐induced BiOtin LIgase diminiSHing. In this method, Bpl1, the only endogenous yeast biotin ligase, is C‐terminally tagged with an auxin‐inducible degron (AID*, Nishimura *et al*, 2009; Morawska & Ulrich, 2013). Therefore, in the presence of auxin and the *Oryza sativa* transport inhibitor response 1 (OsTIR1, Nishimura *et al*, 2009) adaptor protein, the controlled and transient degradation of this essential enzyme ensues, leading to a reduction in biotinylation of its substrates.

**Figure EV1:**
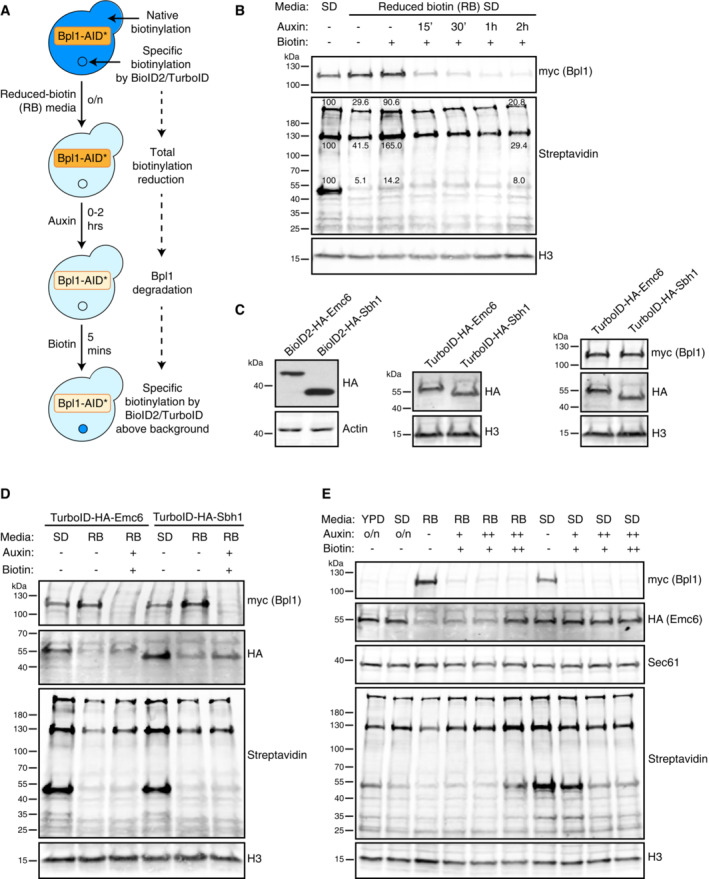
Optimising the conditions used for ABOLISH Schematic of the original growth conditions used to test Bpl1‐AID*‐9myc degradation and endogenous biotinylation reduction.Anti‐Myc and streptavidin blots of cells expressing Bpl1‐AID*‐9myc and OsTIR1 grown overnight and back‐diluted for ~ 4 h in regular synthetic (SD) or reduced biotin (RB) media. To the cells grown in RB media, auxin was either omitted (−) or added 15 min, 30 min, 1 h or 2 h prior to harvesting. Similarly, biotin was either omitted (−) or added 5 min prior to harvesting. Band intensities for the most prominently endogenously biotinylated proteins in lanes 1, 2, 3 and 7 were quantified using Fiji software.Anti‐HA blots confirming the expression of BioID2/TurboID‐HA‐tagged Emc6 and Sbh1. An anti‐Myc blot was included for the strains containing the ABOLISH system.Western blot analysis of cells expressing either TurboID‐HA‐Emc6 or TurboID‐HA‐Sbh1 together with the ABOLISH system. Cells were grown in SD or RB media as in (B). Auxin and biotin were either omitted (−) or added (+) 1.5 h and 30 min, respectively, before harvesting.Western blot analysis of cells expressing TurboID‐HA‐Emc6 and the ABOLISH system. Cells were grown in several different conditions: (i) overnight and back‐diluted in rich media (YPD) containing auxin; (ii) overnight and back‐diluted in regular SD containing auxin; (iii) overnight and back‐diluted in RB media with auxin and biotin either omitted (−), added for 1.5 h and 30 min, respectively, before harvesting (+), or added for the entire duration of the back‐dilution (++); (iv) overnight and back‐diluted in regular SD with auxin and biotin treatments as in (iii). Sec61 was used as an untagged ER membrane protein control. Data information: For panels B–E, H3 (histone H3) or Actin was used as loading controls. Back‐dilution times were all ~ 4 h.

Previously, many of the PL experiments using biotin ligases in yeast aimed to reduce the endogenous biotinylation by preculturing cells in reduced biotin (RB) media (Jan *et al*, 2014; Costa *et al*, 2018; Dahan *et al*, 2022). We therefore tested this condition followed by auxin addition to induce Bpl1 degradation and finally treatments with a biotin pulse (illustrated in Fig EV1A). This demonstrated that RB media alone was not an optimal condition since the initial dramatic reduction in endogenous biotinylation levels was rapidly reversed upon addition of free biotin (Fig EV1B), highlighting the limitations of this method for reducing background biotinylation. Importantly, this reversal was not observed if auxin was used to deplete Bpl1‐AID*‐9myc, proving that ABOLISH can achieve a more complete reduction in background biotinylation noise.

Next, we wanted to track whether ABOLISH could indeed increase the sensitivity of detection when using exogenous, promiscuous, biotin ligase fusions. To do that, we chose a complex for which we could follow both stable and transient PPIs: the most recently characterised ER‐resident insertase; the ER membrane protein complex, EMC (Guna *et al*, 2018). This highly‐conserved machinery is composed of eight subunits (Emc1‐7 & Emc10) in yeast (Jonikas *et al*, 2009) and 10 (EMC1‐10) in humans (Christianson *et al*, 2012). Since its discovery as an insertase for moderately hydrophobic tail‐anchor (TA) proteins (Guna *et al*, 2018; Volkmar *et al*, 2019), it has also been found to insert multi‐pass transmembrane domain (TMD)‐containing proteins into the ER (Chitwood *et al*, 2018; Shurtleff *et al*, 2018; Tian *et al*, 2019; Bai *et al*, 2020; Miller‐Vedam *et al*, 2020; O'Donnell *et al*, 2020; Pleiner *et al*, 2020). Furthermore, it is required for the biogenesis of single‐pass TMD proteins, which do not contain a signal peptide (also known as type III membrane proteins, O'Keefe *et al*, 2021) and a subset of TMD proteins, which traffic from the ER to lipid droplets (LD) (Leznicki *et al*, 2021). To this end, it has a wide, and not yet fully characterised, substrate range and a clear set of stable interactions that we can track.

To compare the various biotin ligases in their capacity to label both stable and transient interactions, and to evaluate whether ABOLISH could enhance the detection of these labelled proteins, we tagged Emc6 at its N‐terminus with either BioID2‐HA or TurboID‐HA (Fig 1C). A third strain expressing both TurboID‐HA‐Emc6 and the ABOLISH system was also generated, along with three control strains in which Sbh1, rather than Emc6, was tagged. Sbh1 was selected as a control to compare against Emc6 primarily because it is an ER membrane protein of similar abundance (Weill *et al*, 2018) whose N‐terminus (used for tagging) also faces the cytosolic side of this organelle. Furthermore, it is part of the SEC machinery required for translocation across the membrane and is therefore functionally comparable, yet distinct from, the EMC. Hence, the comparison between these samples should lead to the discovery of specific EMC interactors. All promiscuous biotin ligase tags were preceded by the constitutive, moderate, *CYC1* promoter to ensure that we do not dramatically overexpress our proteins leading to false positives, and all tagged proteins ran at their expected molecular weights as determined by SDS–PAGE (Fig EV1C).

Surprisingly, we found that overnight growth in RB media resulted in a decrease in the amount of TurboID‐HA‐tagged proteins (Fig EV1D); thus negating the signal‐to‐noise advantage conferred by ABOLISH. To uncover a condition where the TurboID‐tagged protein levels are not reduced but endogenous biotinylation levels are, we tested several different parameters including: overnight growth in either regular YPD or SD media containing auxin; and growth in either RB or regular SD media with auxin and biotin addition at different time‐points (Fig EV1E; see legend for time‐point details). We found that overnight treatment with auxin in SD media (Fig EV1E, 2^nd^ lane) resulted in the strongest background biotinylation reduction without a loss in TurboID‐HA‐Emc6. The requirement for this relatively lengthy auxin treatment is rationalised by the long half‐lives (> 10 h, Christiano *et al*, 2014) of endogenously biotinylated proteins. Interestingly, the abundance of the ER translocon, Sec61, remained constant independent of the conditions tested. This suggests that the loss of TurboID‐tagged proteins triggered by biotin depletion (in RB media) may be a regulatory adaptation to biotin starvation, rather than general protein degradation from the ER. We then calibrated the length of time for biotin treatment that would ensure sufficient labelling material and time for true PPI events to be captured by TurboID without increasing background biotinylation (Fig 1D). We found that even 4 h of exogenous biotin addition did not negate the effect of the auxin‐induced depletion of endogenous biotinylated proteins and therefore this growth pipeline (using regular media and not RB media; illustrated in Fig 1E) was adopted for future ABOLISH experiments. These data collectively demonstrate that the ABOLISH method can be harnessed to reduce background biotinylation ‘noise’, paving the way for enhanced signal detection from exogenous proximity‐labelling enzymes.

### Comparing three biotin ligase systems identifies their ability to uncover both stable and transient protein–protein interactions by LC–MS/MS

**Figure 2:**
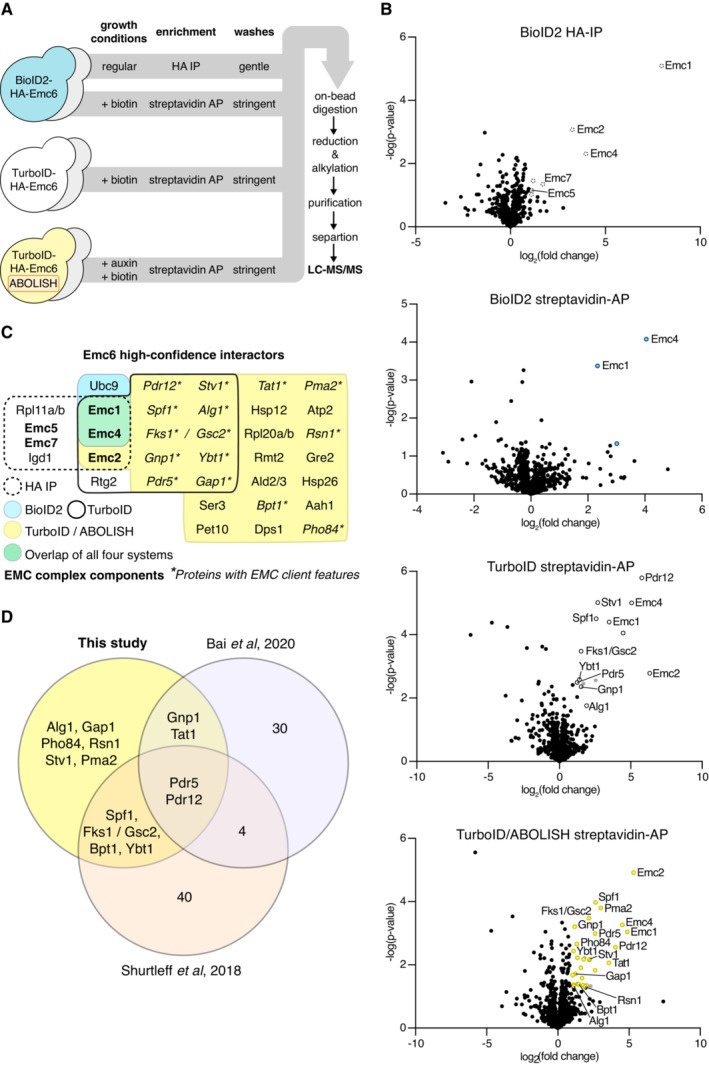
Finding stable and transient Emc6 interactors by a multi‐faceted LC–MS/MS approach Schematic of the workflow used for LC–MS/MS sample preparation of the BioID2‐HA‐Emc6, TurboID‐HA‐Emc6 and TurboID‐HA‐Emc6/ABOLISH strains and their control counterparts (tagged‐Sbh1, depicted as grey yeast). All strains were prepared in biological triplicate.Volcano plots for each of the four distinct LC–MS/MS experiments highlighted in Fig 2A showing ‐log(*P‐*value) against log_2_(fold‐change) for all proteins identified in the appropriate Emc6/Sbh1 samples. Proteins enriched in the Emc6 samples are colour/pattern‐coded as follows: dotted outline for HA‐IP of BioID2‐HA‐Emc6; blue fill for streptavidin‐AP of BioID2‐HA‐Emc6; black, solid outline for streptavidin‐AP of TurboID‐HA‐Emc6; and yellow fill for streptavidin‐AP of TurboID‐HA‐Emc6/ABOLISH. Grey dots represent proteins that passed the *P*‐value and fold‐change criteria but were only identified by one peptide. Only EMC complex components or proteins with EMC client features are named.High‐confidence protein identifications of Emc6 determined by: HA‐IP of BioID2‐HA‐Emc6; streptavidin‐AP of BioID2‐HA‐Emc6; streptavidin‐AP of TurboID‐HA‐Emc6; and streptavidin‐AP of TurboID‐HA‐Emc6/ABOLISH. Colour/pattern coding is as described in (B). EMC complex members and proteins with classical features of EMC substrates are marked in bold and with asterisks, respectively.Overlap between the proteins highlighted with asterisks in (C) and yeast EMC clients found by two independent studies (Shurtleff *et al*, 2018; Bai *et al*, 2020).

While IPs of epitope‐tagged proteins enrich for stable interactors (in this case EMC complex components), streptavidin APs should capture transient interactions labelled by exogenous biotin ligases (in this case clients inserted into the ER by the EMC or regulators of the EMC) and a subset of stable ones; depending on the protein topology and accessibility of K residues on the same side of the membrane. To directly compare the type of interactions that we can identify we analysed, either by HA‐IP or streptavidin‐AP, strains expressing either BioID2‐HA‐Emc6, TurboID‐HA‐Emc6, or TurboID‐HA‐Emc6 on the background of the ABOLISH system (Fig 2A).

**Figure EV2:**
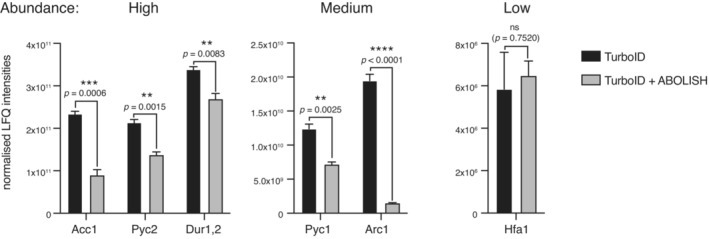
Quantitation of ABOLISH in LC–MS/MS Bar graphs showing the normalised label‐free quantification (LFQ) intensities of endogenously biotinylated proteins, with and without ABOLISH, as measured by LC–MS/MS from biological triplicates. Proteins are grouped into either high, medium or low abundance to enable clear visualisation of the differences. Shown are the standard error of the mean (SEM) and *P*‐values from two‐tailed unpaired *t*‐tests demonstrating significance or not (ns).

Although we had previously observed the reduced background conferred by ABOLISH using Western blot (Fig 1), we first used our MS data to understand the impact of this system on proteomic experiments. We compared the intensities of the six known endogenously biotinylated proteins (Fig 1B) measured by LC–MS/MS from the strains subject to streptavidin‐AP expressing TurboID‐HA‐Emc6 either with or without ABOLISH (Fig EV2). Consistent with our earlier observations (Fig EV1B), Arc1 was the most sensitive to ABOLISH; however, all other endogenously biotinylated proteins were also strongly and significantly reduced, with the exception of Hfa1, the levels of which did not change. Since Hfa1 abundance is very low compared with the other proteins, its insensitivity to ABOLISH has less impact on the background noise. Altogether, endogenously biotinylated proteins made up nearly half (~ 47%) of the total intensity from all proteins identified by LC–MS/MS (as measured by intensity‐based absolute quantification (iBAQ)) from streptavidin‐AP of the TurboID‐HA‐Emc6 strain without ABOLISH. Implementing ABOLISH decreased this number to ~ 27%, demonstrating how much proteomic ‘bandwidth’ is freed up by this approach thus increasing the probability of *bona fide* interactor identification.

Next, we wanted to identify the high‐confidence protein interactors from the different LC–MS/MS runs (Fig 2A). To do this, Emc6 replicate samples were compared with their Sbh1 control counterparts to find high‐confidence protein identifications (Dataset EV1 and via PRIDE, PXD033348). These were defined by the following criteria: a *P*‐value of ≤ 0.05 (streptavidin samples) or ≤ 0.1 (HA samples); a fold‐change of ≥ 2; and identification by two or more unique peptides (Fig 2B). From the HA‐IP, as expected, several EMC complex components satisfied these requirements (Fig 2B and C; dotted outline). From the streptavidin‐AP samples, only three high‐confidence hits were found by BioID2, two of which were Emc1 and Emc4 (Fig 2B and C; blue fill). This confirms that although BioID2 is able to label *bona fide* interactors, its capacity is limited likely due to its relatively low catalytic activity at 30°C (Kim *et al*, 2016). On the contrary, 13 high‐confidence identifications were made by TurboID (Fig 2B and C; black, solid outline). Looking at stable interactors, Emc2 was found in addition to Emc1 and Emc4, already hinting at increased labelling functionality relative to BioID2. Importantly, the luminal EMC component, Emc7, was not found, indicating that there was no postlysis biotinylation by the cytosolic‐facing TurboID. Most encouragingly, of the remaining 10 putative interactors, nine had membrane protein features classically associated with EMC clients, suggesting an increased capacity to uncover transient interactions (Fig 2C; asterisks). Eight of these are multi‐pass TMD secretory pathway proteins, and the remaining protein (Alg1) is a lipid‐droplet (LD) protein with a single N‐terminal TMD—a characteristic recently demonstrated to define EMC‐dependence (Leznicki *et al*, 2021).

Furthermore, incorporating the ABOLISH system enabled the detection of even more putative interactors labelled by TurboID (Fig 2B and C; yellow fill). The overlap between both TurboID strategies was very large with the same stable interactions and all nine candidate substrates being found. Another 16 high‐confidence identifications were made, five of which were secretory pathway multi‐pass TMD proteins (Fig 2C; asterisks). Comparing our list of putative substrates to published yeast EMC client data found by ribosome profiling (Shurtleff *et al*, 2018) and proteomic analysis of WT vs *EMC3* KO cells (Bai *et al*, 2020), revealed that eight out of the 14 identified had previously been found in either study (Fig 2D) supporting the validity of our transient PPI discovery. To our knowledge, this is the first time TurboID has been successfully used in baker's yeast, and our data demonstrate that it labels both stable and transient PPIs. In addition, the ABOLISH system enhances the capacity to detect TurboID‐labelled interactors.

### Validating new EMC substrates using genetic tools and a natively‐expressed pairwise biotinylation method

**Figure 3:**
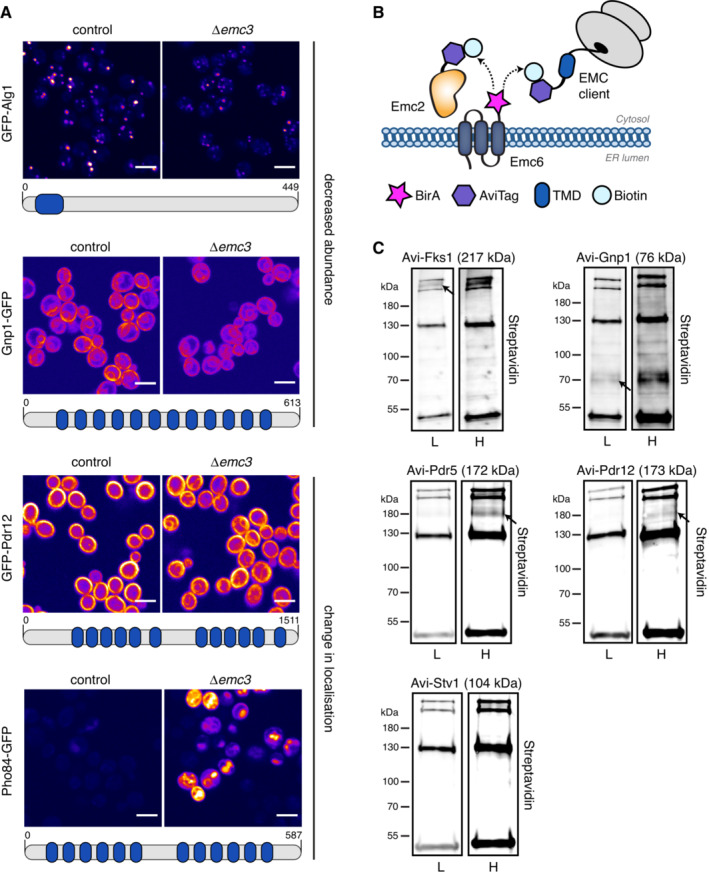
Verifying new EMC clients using microscopy and BirA‐AviTag combined with ABOLISH Fluorescence microscopy images of control or Δ*emc3* strains containing either Alg1 N′ tagged with GFP, Gnp1 C′ tagged with GFP, Pdr12 N′ tagged with GFP or Pho84 C′ tagged with GFP. For the Gnp1‐GFP strain, the quantified signal depletion was ~ 10% (*P* < 0.0001). Topological schematics feature beneath each respective image, with predicted TMDs highlighted in blue (Weill *et al*, 2019). All images are from two independent biological repeats and are digitally coloured using the ‘fire’ lookup table (LUT) on Fiji. The same contrast settings were used for each image pair. Scale bar = 5 μm.Depiction of the BirA‐AviTag system where BirA‐tagged Emc6 can biotinylate stably‐interacting AviTag‐EMC components (such as Emc2) and transient AviTag‐substrates.Streptavidin blots of diploid strains expressing either AviTag‐Fks1, ‐Gnp1, ‐Pdr5, ‐Pdr12 or ‐Stv1 (serves as negative control) together with BirA‐Emc6, grown in the conditions described in Fig 1E. For each strain, blots showing both the ‘lower’ (L) and ‘higher’ (H) contrast settings are shown side‐by‐side. The expected molecular weight in kDa for each tested protein including AviTag is written in parentheses after the protein name. The arrows indicate the bands corresponding to the molecular weight of each AviTagged protein.

**Figure EV3:**
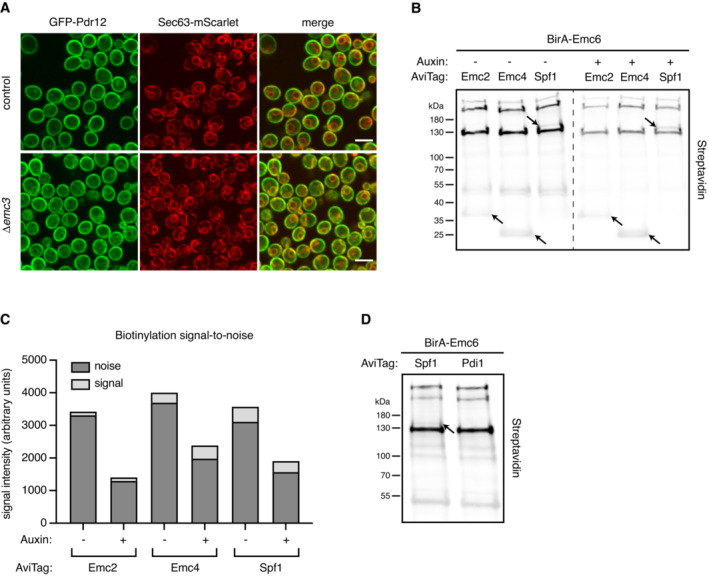
Contribution of the ABOLISH system to noise reduction in BirA‐AviTag blotting Fluorescence microscopy images of control and Δ*emc3* strains containing Pdr12 N′ tagged with GFP and Sec63 C′ tagged with mScarlet. Sec63 localisation was unchanged upon loss of Emc3 and no significant difference was found in signal intensity (*P* = 0.1156). Scale bar = 5 μm.Streptavidin blot of diploid strains expressing either AviTag‐Emc2, ‐Emc4, or ‐Spf1 together with BirA‐Emc6, grown in media with or without auxin. 15 μg of whole‐cell lysate from each sample was loaded onto the gel. The arrows indicate the bands corresponding to the molecular weight of each of the AviTagged proteins.Quantitation of the streptavidin signal from endogenously biotinylated proteins (noise) and biotinylated AviTagged proteins (signal) show a reduction in the background noise by ~half when ABOLISH is activated.Streptavidin blot of diploid strains expressing either AviTag‐Spf1 or ‐Pdi1 together with BirA‐Emc6, grown in media containing auxin. The arrow indicates the band corresponding to the molecular weight of the AviTagged protein.

The similarity between our list of candidate EMC clients and published datasets (Fig 2D) strongly suggested that TurboID‐mediated proteomics, both with and without ABOLISH, identified *bona fide* EMC substrates. Such substrates should be affected by loss of the complex, and indeed, it was previously shown that the abundance and/or localisation of true EMC substrates changes upon Emc3 loss (Bai *et al*, 2020). We therefore deleted *EMC3* on a selection of our candidates and observed a strong reduction in the abundance of GFP‐Alg1 (Fig 3A, top panel) and, to a lesser, but still significant, extent, Gnp1‐GFP (Fig 3A, 2^nd^ panel). Deletion of *EMC3* also changed the localisation of GFP‐Pdr12 relative to the control strain (Fig 3A, 3^rd^ panel). Pdr12 is a plasma membrane (PM) ATP‐binding cassette (ABC) transporter, which first requires insertion into the ER before trafficking to its final destination. Therefore, the accumulation of Pdr12 on the ER (shown by colocalisation with ER‐resident Sec63; Fig EV3A) in the Δ*emc3* strain likely signifies a preinserted population at the ER surface. Interestingly, Pho84‐GFP was also affected by the loss of *EMC3* (Fig 3A, bottom panel). Pho84 is an inorganic phosphate transporter, which traffics along the secretory pathway to the PM. In phosphate‐rich media (used in these experiments) it is known to be internalised and degraded by the vacuole and hence very weakly detectable in the WT images (Petersson *et al*, 1999; Hürlimann *et al*, 2007). *EMC3* deletion, however, led to a strong accumulation of Pho84‐GFP signal at internal membranes. These changes were specific to the proposed clients since localisation and abundance of the ER membrane protein, Sec63, were not affected by perturbation of the EMC (Fig EV3A). Collectively, these functional assays support these proteins as newly‐validated clients of the EMC complex.

More broadly, however, verifying transient interactions is, in itself, a challenging task as methods to validate PPIs (such as co‐IP) are again optimised for very stable interactions. We therefore used a parallel biotinylation approach involving the BirA biotin ligase, which specifically biotinylates the AviTag sequence (Cronan, 1990; Beckett *et al*, 1999). In this setup, even transient protein–client interactions can be assayed *in vivo* and at physiological expression levels. To do this, a haploid strain expressing a BirA‐tagged protein (e.g. Emc6) under its native promoter is mated with a haploid strain of the opposite mating type, which expresses a potential interactor N‐terminally tagged with AviTag (also under native promoter control). The diploid strains can then be analysed for the appearance of a streptavidin‐positive band that proves that BirA came sufficiently close to the AviTag (illustrated in Fig 3B). Initially, well‐characterised and previously validated interactors were selected to test the utility and feasibility of this validation method: hence strains expressing either AviTag‐Emc2, ‐Emc4, or ‐Spf1 (Jonikas *et al*, 2009; Shurtleff *et al*, 2018) were crossed with the BirA‐Emc6 strain. To ensure the best signal‐to‐noise ratio in this setup, we integrated the ABOLISH system into these strains, and this clearly reduced the background signal and made the assay even cleaner (Fig EV3B and C). The ABOLISH system is therefore critically important for: lower abundance proteins; more transient interactions; proteins that are less efficiently biotinylated; or proteins whose molecular weight is similar to that of endogenously biotinylated proteins. Hence ABOLISH can also extend the dynamic range of BirA‐AviTag for pairwise, gel‐based assays.

This assay is highly specific since it did not falsely report an interaction for the highly abundant ER‐resident protein, Pdi1 (Fig EV3D), in contrast to the clearly visible bands for Emc2, Emc4 and Spf1 (Fig EV3B and C). This made us confident that our assay reveals *bona fide* interactions *in vivo*. Indeed, Fks1, an interactor found by TurboID (Fig 2B and C) and a known EMC substrate (Shurtleff *et al*, 2018), was readily detected by streptavidin blot using the BirA‐AviTag/ABOLISH system (Fig 3C). The AviTagged amino acid permease, Gnp1, was similarly easy to detect. Some clients required a higher contrast setting to be visualised. However, both AviTag‐Pdr5 and AviTag‐Pdr12 produced clear streptavidin‐reactive bands compared with AviTag‐Stv1, which did not produce a detectable Emc6 interaction (Fig 3C). Collectively, these data highlight the power of the BirA‐AviTag/ABOLISH system for providing a rare, *in vivo* ‘snapshot’ of the transient interactions between the EMC insertase and both previously‐confirmed (Shurtleff *et al*, 2018; Bai *et al*, 2020) and newly‐validated (Fig 3A and C) substrates. More broadly it serves as a rapid, systematic pipeline for the validation of TurboID interactomes.

### Generating a biotinylation toolkit: a collection of five full‐genome libraries to facilitate high‐throughput protein–protein interaction discovery

Through studying and comparing the promiscuous biotin ligases that can be used in yeast, we have demonstrated that TurboID, especially when combined with the ABOLISH system, serves as an unbiased tool to efficiently label stable and transient functional interactors in *S. cerevisiae*. This in turn can lead to the discovery of novel protein‐machinery substrates, as highlighted for the EMC (Figs 2 and 3), and regulators. We have also demonstrated the suitability of the BirA‐AviTag technology for assaying and validating native pairwise interactions and the capacity of this sensitive methodology to highlight even transient interactions.

**Figure 4:**
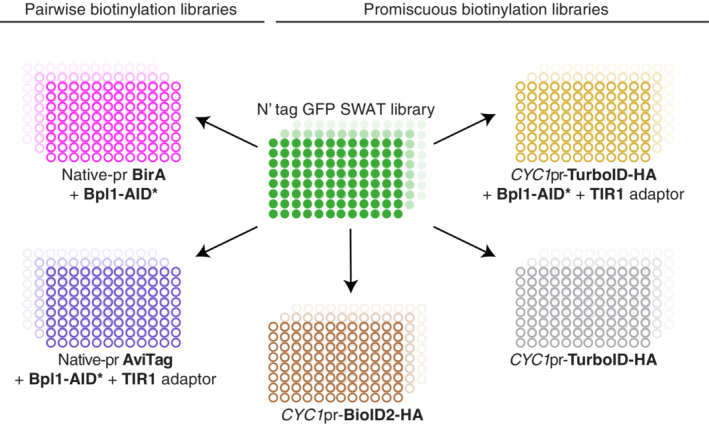
Generating five full‐genome libraries using the SWAT method Schematic representation of the creation of new library collections. The original N′ tag GFP SWAT library was used to generate five full‐genome libraries using an automated process of mating, selection, sporulation and SWATing (see Materials and Methods for details). All promiscuous biotin ligase libraries have a *CYC1*pr and synthetic targeting signals upstream of the BioID2‐HA/TurboID‐HA, whereas the pairwise biotinylation libraries have BirA and AviTag downstream of the native promoter and targeting sequences. The AviTag/ABOLISH and TurboID‐HA/ABOLISH libraries express the OsTIR1 adaptor protein and Bpl1‐AID*‐6HA or Bpl1‐AID*‐9myc, respectively. The BirA library includes only Bpl1‐AID*‐9myc.

To truly harness the power of these biotinylation tools and make them widely applicable, we created whole‐proteome collections of yeast strains (also called libraries) using our recently developed approach for yeast library generation called SWAp Tag (SWAT) (Yofe *et al*, 2016; Meurer *et al*, 2018; Weill *et al*, 2018). This approach allows us to take an initial library and swap its tag to any one of our choice. Therefore, using the N′ GFP SWAT library and accompanying SWAT protocol (Yofe *et al*, 2016; Weill *et al*, 2018) we generated five whole‐genome libraries (Fig 4). In the first two, each strain encodes one yeast protein fused at its N′ to a TurboID‐HA tag expressed under the control of a medium‐strength constitutive *CYC1* promotor (Yofe *et al*, 2016; Weill *et al*, 2018) with, or without, the ABOLISH system. The third library is an N′ tag *CYC1*pr‐BioID2‐HA collection. All three libraries contain generic N′ localisation signals (signal peptides (SPs) and mitochondrial targeting signals (MTS) (Yofe *et al*, 2016; Weill *et al*, 2018)) where required. The last two full‐genome libraries express N‐terminally tagged proteins with either BirA or AviTag under their endogenous promoters and native N′ localisation signals, and the ABOLISH system is also integrated. In addition to PPI validation (as shown above) these libraries can, of course, be used for hypothesis‐driven interrogation of interactions between any two proteins of interest.

**Figure EV4:**
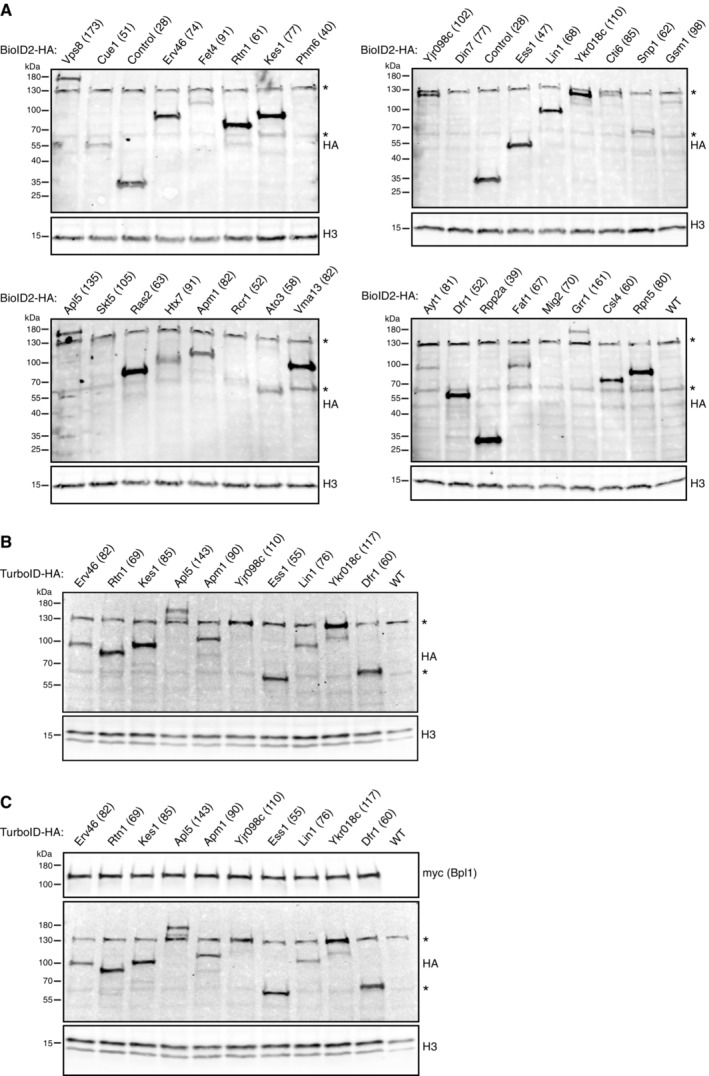
Quality control checks on biotinylation toolkit libraries A–CWestern blot analysis for selected strains from the BioID2‐HA (A) TurboID‐HA (B) and TurboID‐HA/ABOLISH (C) libraries. For the BioID2‐HA library, Phm6, Din7, Skt5, Rcr1 and Mig2 all have a low endogenous expression (relative intensity values of ≤ 27, Weill *et al*, 2018), which likely explains why they were not readily detectable. ‘Control (28)’ refers to BioID2‐HA not tagged to any protein, and ‘WT’ denotes lysate ran from the BY4741 laboratory strain to highlight nonspecific bands; the most prominent of which are marked with an asterisk. For all panels, H3 (histone H3) is used as a loading control. The expected molecular weight in kDa for each tested protein including their tag is written in parentheses after the protein name.

All newly‐generated libraries were subject to strict quality control checks (see Methods). Furthermore, a number of new library strains were selected and subject to SDS–PAGE analysis to confirm both protein expression and that the new tag had recombined in‐frame during the SWAT process. This was demonstrated to be the case for the BioID2‐HA (Fig EV4A), TurboID‐HA (Fig EV4B) and TurboID‐HA/ABOLISH (Fig EV4C) libraries. Hence these represent five high‐coverage yeast libraries that will be freely distributed to enable high‐throughput exploration, discovery and validation of stable and transient interactions throughout the yeast proteome.

## Discussion

Our work demonstrates the power of proximity biotin labelling tools for the exploration of both stable and transient protein interactions in yeast. We also show, for the first time, the utility of TurboID in this model organism. The combination of TurboID‐based proximity‐labelling and standard HA‐IPs, which can be performed from the same sample thanks to our tag design, is the most powerful approach for finding an extensive repertoire of stable and transient PPIs. Surprisingly, we found that previously‐utilised protocols of growth in low biotin media to reduce endogenous biotinylation levels (Jan *et al*, 2014) did not negate the rapid rebiotinylation of endogenous substrates, even upon a short biotin pulse, and also resulted in the downregulation of TurboID‐tagged proteins. This highlights the advantage of using our ABOLISH system, which is designed to increase streptavidin‐specific signal‐to‐noise through controlled Bpl1 degradation. The reduction in endogenous biotinylation levels leads to lower sample complexity and hence increased sensitivity—indeed, more interactors were found by TurboID when it was coupled to the ABOLISH system, despite the fact that advanced MS instrumentation was used. Furthermore, the ABOLISH approach should be applicable to several model organisms provided the endogenous biotinylation machinery has been identified and known to tolerate tagging. Naturally, model systems expressing high levels of background biotinylation would stand to gain the most from implementing ABOLISH.

The multi‐subunit EMC (Jonikas *et al*, 2009; Christianson *et al*, 2012) has recently been characterised as an ER membrane insertase (Guna *et al*, 2018), and as such, several of its substrates have been elucidated, particularly in human cells (Chitwood *et al*, 2018; Guna *et al*, 2018; Shurtleff *et al*, 2018; Tian *et al*, 2019; Volkmar *et al*, 2019; Leznicki *et al*, 2021; O'Keefe *et al*, 2021). Using EMC as a test case for TurboID utility in baker's yeast, we discovered six new putative substrates of this complex, in addition to reproducing interactions previously identified. In the past, EMC substrates were uncovered using *in vitro* assays (Chitwood *et al*, 2018; Guna *et al*, 2018; Leznicki *et al*, 2021; O'Keefe *et al*, 2021), labour‐intensive ribosome profiling (Shurtleff *et al*, 2018), or proteomic profiling comparing control vs ΔEMC cells (Shurtleff *et al*, 2018; Tian *et al*, 2019; Volkmar *et al*, 2019; Bai *et al*, 2020). While loss‐of‐function studies have clearly proven useful, they suffer from both false negatives (from the presence of backup systems, Ihmels *et al*, 2007) and false positives (resulting from off‐target effects). Endogenous labelling of transiently interacting substrates *in vivo* can therefore offer a complementary approach to validate direct interactions and facilitate protein substrate discovery.

Of the six new candidate yeast EMC substrates that we identified, Alg1 stands out as unique. It is a highly‐conserved and essential mannosyltransferase localised to LDs (Krahmer *et al*, 2013) and possesses a single N‐terminal hydrophobic TMD. This type of substrate was only recently established to require the EMC for its biogenesis in humans (Leznicki *et al*, 2021). Notably, the free energy difference (ΔG, Hessa *et al*, 2007) for the TMD of Alg1 is −2.097, highly consistent with the ΔG values observed for the TMDs of human EMC‐dependent LD proteins (Leznicki *et al*, 2021).

In addition to Alg1, we also found that Gnp1, Pdr12 and Pho84 behave as substrates. Both Gnp1 and Pdr12 were previously flagged as putative yeast EMC substrates (Shurtleff *et al*, 2018; Bai *et al*, 2020), however, remained unvalidated. Additionally, it seems that EMC‐dependence for these proteins is conserved throughout evolution, with the levels of SLC7A1 and ABCA3 (human homologues of Gnp1 and Pdr12, respectively, Fenech *et al*, 2020) reduced in EMC KO cells (Tang *et al*, 2017; Tian *et al*, 2019). Interestingly, a physical association between EMC3 and ABCA3 was also reported (Tang *et al*, 2017), supporting our evidence for an EMC‐Pdr12 interaction. Lastly, Pho84 was uniquely found by TurboID + ABOLISH (Fig 2B and C) and was not previously identified as a putative EMC substrate. Interestingly, however, the phosphate transporter paralogs Pho87 and Pho90 were identified as EMC client candidates (Shurtleff *et al*, 2018; Bai *et al*, 2020), suggesting a role for the EMC in phosphate uptake.

Naturally, not all putative substrates were identified or confirmed using proximity biotinylation methods. There are several aspects of each method that should therefore be thought of when choosing which of the libraries to utilise for PPI detection. For example, BirA specifically biotinylates AviTag and both modules must be proximal in space and on the same side of the membrane for biotinylation to occur. TurboID, on the contrary, has more labelling opportunities as it can biotinylate any topologically available lysine residue within an interactor sequence. Other differences to bear in mind include the fact that the BirA‐AviTag assay is carried out in diploid cells; unlike the haploid TurboID‐expressing strains. Also, the TurboID‐tagged proteins are under the control of the constitutive *CYC1* promoter and generic N′ localisation signals. This is in contrast to the proteins tagged with BirA/AviTag, which are under the control of their native promoter and localisation signals. These native features provide much more physiological conditions, even though some low‐abundance proteins may be less easy to detect. Finally, as opposed to proteomic‐based approaches, the BirA‐AviTag system serves as a much cheaper and easy‐to‐use method requiring no specialist equipment.

In addition, despite the clear benefits of the ABOLISH system, we generated a TurboID ‘only’ library for instances where reduced biotinylation of endogenous substrates of Bpl1 or the addition of auxin itself may interfere with the proteins being studied. For example, it has been shown that auxin inhibits the TORC1 pathway (Nicastro *et al*, 2021). Similarly, a BioID2 library was also included as part of our toolkit since it has already been adopted by the yeast community (Opitz *et al*, 2017; Singh *et al*, 2020). It is a smaller ‘tag’ compared with TurboID (27 kDa vs. 35 kDa) and is known to have higher activity at higher temperatures; thus may prove useful for heat‐shock experiments, for example.

We believe that the unique properties of the promiscuous and pairwise biotinylation machineries make the combination of both approaches the most powerful tool for stable, and even more so, transient PPI discovery and validation in baker's yeast. Our whole‐genome libraries and accompanying sample preparation protocols provide a broad resource for functional proteome exploration. Altogether, we present a complete biotinylation toolkit to enable high‐throughput interaction profiling in baker's yeast. This should now support the systematic characterisation of new protein functions, definition of substrate ranges for protein machineries, elucidation of signalling pathways and tracking of dynamic organellar processes.

## Materials and Methods

### Reagents and Tools table

**Table:** 

Reagent/Resource	Reference or Source	Identifier or Catalog Number
**Experimental Models**		
*List cell lines, model organism strains, patient samples, isolated cell types etc. Indicate the species when appropriate*		
*Saccharyomyces cerevisiae* strains (S288C background)	Schuldiner lab	Dataset EV2
**Recombinant DNA**		
*Indicate species for genes and proteins when appropriate*		
Tagging, knock‐out and SWAT plasmids	Schuldiner lab	Dataset EV3
**Antibodies**		
*Include the name of the antibody, the company (or lab) who supplied the antibody, the catalogue or clone number, the host species in which the antibody was raised and mention whether the antibody is monoclonal or polyclonal. Please indicate the concentrations used for different experimental procedures*		
Mouse anti‐HA	BioLegend	Cat # 901502
Rabbit anti‐myc	Abcam	Cat # ab9106
Rabbit anti‐Histone H3	Abcam	Cat # ab1791
Rabbit anti‐Sec61	A gift from Matthias Seedorf and Marius Lemberg	N/A
Mouse anti‐Actin	Abcam	Cat # ab8224
Goat anti‐rabbit IgG 800	Abcam	Cat # ab216773
Goat anti‐mouse IgG 800	Abcam	Cat # ab216772
Goat anti‐rabbit IgG 680	Abcam	Cat # ab216777
Goat anti‐mouse IgG 680	Abcam	Cat # ab216776
**Oligonucleotides and sequence‐based reagents**		
*For long lists of oligos or other sequences please refer to the relevant Table(s) or EV Table(s)*		
PCR primers	This study	Dataset EV4
**Chemicals, enzymes and other reagents**		
*e.g. Drugs, peptides, recombinant proteins, dyes etc*.		
Auxin (3‐indoleacetic acid)	Sigma	Cat # I3750
D‐Biotin	Supelco	Cat # 47868
Nourseothricin (NAT)	Quimigen	Cat # AB‐102‐25G
Hygromycin (HYG)	Formedium	Cat # HYG5000
G418	Formedium	Cat # G4185
5‐fluoro‐orotic acid monohydrate (5‐FOA)	Formedium	Cat # 5FOA05
Protease inhibitors	Merck	Cat # 539134
Benzonase	Sigma	Cat # E1014
Digitonin	Sigma	Cat # D141
Streptavidin magnetic beads	Sigma	Cat # 28‐9857‐99
Protein G magnetic beads	Sigma	Cat # 28‐9513‐79
SDS	BioRad	Cat # 1610418
Urea	Sigma	Cat # U5378
Iodoacetamide (IAA)	Sigma	Cat # I6125
Dithiothretol (DTT)	Sigma	Cat # D9779
Trypsin	Promega	Cat # V5111
Oasis desalting column	Waters	Cat # 186001828BA
C18 trapping column	Waters	Cat # 186008821
Fluorescent streptavidin	Thermo Scientific	Cat # S11378
**Software**		
*Include version where applicable*		
MaxQuant v1.6.6.0	maxquant.org/maxquant/	
Perseus v1.6.2.3	maxquant.org/perseus/	
ScanR Analysis Software v3.2.0.0	Olympus	
ImageJ/FiJi	Schindelin *et al* (2012)	
Prism v9.3.0	GraphPad Software	
**Other**		
*Kits, instrumentation, laboratory equipment, lab ware etc. that are critical for the experimental procedure and do not fit in any of the above categories can be listed here*		
FastPrep‐24 cell homogeniser	MP Biomedicals	
FastPrep‐24 2ml tubes loaded with Lysing Matix C	MP Biomedicals	Cat # 116912100
Q Exactive HF quadrupole orbitrap mass spectrometer	Thermo Scientific	
RoToR array pinning robot	Singer Instruments	

### Methods and Protocols

#### Yeast strains and plasmids

All yeast strains used in this study are listed in Dataset EV2. Strains were constructed using the lithium acetate‐based transformation protocol (Gietz & Woods, 2002). All plasmids used are listed in Dataset EV3 (see also Longtine *et al*, 1998; Janke *et al*, 2004) and primers were designed with the Primers‐4‐Yeast web tool (https://www.weizmann.ac.il/Primers‐4‐Yeast/, Yofe & Schuldiner, 2014, Dataset EV4). The original SWAT donor strain (yMS2085; Weill *et al*, 2018) was transformed with SWAT donor plasmids encoding BioID2‐HA or TurboID‐HA. SWAT donor strains encoding the ABOLISH system were constructed by C‐terminally tagging Bpl1 with AID*‐9myc and integrating the OsTIR1 adaptor into the *HIS* locus using a PmeI‐cut, OsTIR1‐encoding plasmid (Morawska & Ulrich, 2013; Orgil *et al*, 2015). These strains were transformed with SWAT donor plasmids encoding TurboID‐HA, BirA, or AviTag.

#### Yeast growth

Yeast cells were grown on solid media containing 2.2% agar or liquid media. YPD (2% peptone, 1% yeast extract, 2% glucose) was used for cell growth if only antibiotic selections were required, whereas synthetic minimal media (SD; 0.67% [w/v] yeast nitrogen base (YNB) without amino acids and with ammonium sulphate or 0.17% [w/v] YNB without amino acids and with monosodium glutamate, 2% [w/v] glucose, supplemented with required amino acid) was used for auxotrophic selection. Antibiotic concentrations were as follows: nourseothricin (NAT, Quimigen) at 0.2 g/l; G418 (Formedium) at 0.5 g/l; and hygromycin (HYG, Formedium) at 0.5 g/l. Yeast grown for transformation, protein extraction, or LC–MS/MS analysis was first grown in liquid media with full selections overnight at 30°C and subsequently back‐diluted into YPD/SD media to an OD_600_ of ~ 0.2. Cells were collected after at least one division but before reaching an OD_600_ of 1 and either immediately used for transformation or snap‐frozen for later processing. Cells grown for streptavidin‐AP followed by LC–MS/MS were treated for ~ 18 h with 50 μM biotin as in Roux *et al* (2012), Branon *et al* (2018), Larochelle *et al* (2019), and Singh *et al* (2020). For all ABOLISH experiments, biotin was used at 100 nM with the exception of Fig EV1B, where it was used at 10 nM (Jan *et al*, 2014). For Fig EV1B, D and E, RB media was prepared as specified in (Jan *et al*, 2014). Auxin was used at 1 mM. Times of treatments are specified in the appropriate figure legends.

#### Protein extraction and SDS–PAGE analysis

Cell pellets were resuspended in 200 μl lysis buffer (8 M urea, 50 mM Tris–pH 7.5, protease inhibitors (Merck)). From there on, processing, SDS–PAGE separation, Western blotting and fluorescent‐based imaging was done as described in Eisenberg‐Bord *et al* (2021), with the exception of the HA blot in Fig EV1C. Here, the SDS–PAGE gel was blotted onto PVDF membrane (Millipore) by wet transfer, and imaging was done using X‐ray film (FujiFilm) to detect signal from HRP‐conjugated anti‐mouse secondary antibody (1:7,500, Jackson ImmunoResearch, #111‐035‐003) incubated with ECL substrate (Thermo Scientific). The following antibodies were used for Western blot: anti‐HA (1:1,000, BioLegend, #901502), anti‐Myc (1:3,000, Abcam, #ab9106), anti‐Histone H3 (1:5,000, Abcam, #ab1791), anti‐Sec61 (1:5,000, a kind gift from Matthias Seedorf of Heidelberg University and Marius Lemberg of the University of Cologne), anti‐Actin (1:2,000, Abcam, #ab8224), goat anti‐rabbit IgG H&L 800CW (1:7,500, Abcam, #ab216773) and goat anti‐mouse IgG H&L 680RD (1:7,500, Abcam, #ab216776). Membranes were incubated for 1 h at RT with fluorescent streptavidin (1:10,000, Invitrogen, #S11378) diluted in 2% (w/v) BSA/PBS containing 0.01% NaN_3_ to detect biotinylated proteins.

#### Immunoprecipitation and LC–MS/MS sample preparation

Cell pellets from a total of ~ 5ODs were resuspended in 400 μl lysis buffer (150 mM NaCl, 50 mM Tris–HCl pH 8.0, 5% Glycerol, 1% digitonin (Sigma, #D141), 1 mM MgCl_2_, protease inhibitors (Merck), benzonase (Sigma, #E1014)). The cell suspension was then transferred to a 2 ml FastPrep™ tube (lysing matrix C, MP Biomedicals) and lysis was carried out by 6 × 1 min maximum speed cycles on a FastPrep‐24™ cell homogeniser (MP Biomedicals), with the samples being returned to ice for 5 min between each cycle. Lysates were cleared at 16,000 *g* for 10 min at 4°C, and the supernatant was transferred to a fresh microcentrifuge tube. For HA‐IP, samples were first incubated for 1 h at 4°C with 2 μl of anti‐HA antibody (BioLegend) and then for another hr after adding 30 μl of washed magnetic ProteinG beads (Cytiva). The beads were washed twice with 200 μl of digitonin wash buffer (150 mM NaCl, 50 mM Tris–HCl pH 8.0, 1% digitonin) and then four times in basic wash buffer (150 mM NaCl, 50 mM Tris–HCl pH 8.0) before being incubated with 50 μl elution buffer (2 M urea, 20 mM Tris–HCl pH 8.0, 2 mM DTT and 0.5 μl trypsin (0.5 μg/μl, Promega, #V5111)) per sample for 90 min. The eluate was removed from the beads and collected in a fresh microcentrifuge tube. 50 μl alkylation buffer (2 M urea, 20 mM Tris–HCl pH8.0, 50 mM iodoacetamide (IAA)) was then added to the beads and incubated for 10 min. This buffer was also removed from the beads and combined with the first eluate. Finally, the beads were washed with 50 μl urea buffer (2 M urea, 20 mM Tris–HCl pH 8.0) for another 10 min, and again, the buffer was removed and combined with the above mixture. All elution steps were carried out at room temperature (RT) in the dark with shaking (1,400 rpm). The eluted mixture (150 μl total volume) was incubated overnight at RT in the dark at 800 rpm. The following morning 1 μl 0.25 μg/μl trypsin was added to each sample and incubated for a further 4 h at RT in the dark at 800 rpm. For streptavidin‐AP, the same protocol as for HA‐IP was used with the following changes: (i) 100 μl streptavidin‐conjugated beads (Cytiva) were used per sample and incubated overnight at 4°C; (ii) post‐AP beads were washed twice in 500 μl 2% SDS wash buffer (2% v/v SDS (BioRad, #1610418), 150 mM NaCl, 50 mM Tris–HCl pH 8.0), twice in 500 μl 0.1% SDS wash buffer, then twice in 500 μl basic wash buffer. All washes were 5 min and were carried out at RT on overhead rotator. Following digestion, peptides were desalted using Oasis HLB, μElution format (Waters, Milford, MA, USA). The samples were vacuum dried and stored at −80°C until further analysis.

#### LC–MS/MS settings and analysis

ULC/MS‐grade solvents were used for all chromatographic steps. Each sample was loaded using split‐less nano‐Ultra Performance Liquid Chromatography (10 kpsi nanoAcquity; Waters, Milford, MA, USA). The mobile phase was: (A) H_2_O + 0.1% formic acid, and (B) acetonitrile +0.1% formic acid. Desalting of the samples was performed online using a reversed‐phase Symmetry C18 trapping column (180 μm internal diameter, 20 mm length, 5 μm particle size; Waters). The peptides were then separated using a T3 HSS nano‐column (75 μm internal diameter, 250 mm length, 1.8 μm particle size; Waters) at 0.35 μl/min. Peptides were eluted from the column into the mass spectrometer using the following gradient: 4–30% B over 55 min, 30–90% B over 5 min, maintained at 90% for 5 min and then back to the initial conditions. The nanoUPLC was coupled online through a nanoESI emitter (10 μm tip; New Objective, Woburn, MA, USA) to a quadrupole orbitrap mass spectrometer (Q Exactive HF; Thermo Scientific) using a FlexIon nanospray apparatus (Proxeon). Data were acquired in data‐dependent acquisition (DDA) mode, using a Top10 method. MS1 resolution was set to 120,000 (at 200 *m/z*), mass range of 375–1,650 *m/z* and AGC of 3e6, and maximum injection time was set to 60 ms. MS2 resolution was set to 15,000, quadrupole isolation 1.7 *m/z*, AGC of 1e5, dynamic exclusion of 20 s and maximum injection time of 60 ms.

#### LC–MS/MS raw data processing

Raw data were processed with MaxQuant v1.6.6.0. The data were searched with the Andromeda search engine against the SwissProt *S. cerevisiae ATCC204508/S288c* proteome database (November 2018 version, 6049 entries) in addition to the MaxQuant contaminants database. All parameters were kept as default except: Minimum peptide ratio was set to 1; maximum of 3 miscleavages were allowed; and match between runs was enabled. Carbamidomethylation of C was set as a fixed modification. Oxidation of M, deamidation of N and Q and protein N‐term acetylation were set as variable modifications. The LFQ intensities were used for further calculations using Perseus v1.6.2.3. Decoy hits were filtered out, as well as proteins that were identified on the basis of a modified peptide only. The LFQ intensities were log2‐transformed and only proteins that had at least 2 valid values in at least one experimental group were kept. The remaining missing values were imputed by a random low‐range distribution. Student's *t*‐tests were performed between the relevant groups to identify significant changes in protein levels. The mass spectrometry proteomics data have been deposited to the ProteomeXchange Consortium via the PRIDE (Perez‐Riverol *et al*, 2021) partner repository with the dataset identifier PXD033348.

#### Imaging

Cells were grown overnight in 100 μl SD media (with appropriate amino acid (AA) selections) in a round‐bottomed 96‐well plate (ThermoFisher) at 30°C with shaking. 5 μl of overnight culture was back‐diluted into 95 μl YPD and incubated for ~ 4 h at 30°C with shaking. 50 μl of culture was subsequently transferred to a Concanavalin A (ConA, Sigma, 0.25 mg/ml)‐coated 384‐well glass‐bottomed microscopy plate (Matrical Bioscience) and incubated for 20 min at RT. The media was removed and the cells were washed twice in 50 μl SD‐riboflavin+completeAA prior to being imaged in the same media at RT. Images for the Alg1, Pdr12 and Pho84 panels of Figs 3A and EV3A were obtained using a VisiScope Confocal Cell Explorer system (Visitron Systems) coupled to an inverted IX83 microscope (Olympus), a CSU‐W1‐T1 50μm spinning disk scanning unit (Yokogawa) and an Edge sCMOS camera (PCO) controlled by VisiView software (V3.2.0, Visitron Systems). A 60x oil objective was used (NA = 1.42, Olympus) together with a GFP filter EX470/40 nm, EM525/50 nm (Chroma) and a 100 mW 488 nm laser (Visitron Systems). Images for the Gnp1 panel of Fig 3A were obtained using a SpinSR system (Olympus) coupled to a CSUW1‐T2SSR spinning disk scanning unit (Yokogawa) and an ORCA‐Flash 4.0 CMOS camera (Hamamatsu). A 60x air objective was used (NA = 0.9, Olympus) together with a GFP filter EX470/40 nm, EM525/50 nm (Chroma) and a 100 mW 488 nm laser system (Coherent OBIS LX). All images are single‐focal planes. Fiji was used for image inspection and brightness adjustment (Schindelin *et al*, 2012). For quantitation (Gnp1‐GFP in Fig 3A, Sec63‐mScarlet in Fig EV3A), the images were first processed using the ScanR Analysis software (V3.2.0.0, Olympus). Processing used a neural network virtual channel module to segment the image (transmitted channel only) to cells using the intensity object segmentation module. Next, noise and objects that were poorly segmented were removed based on their area and circularity factor, and for each cell, the mean signal intensity in either the 488 or 561 channel was measured. The following Python script was used for data analysis: https://github.com/Maya‐Schuldiner‐lab/Analysis‐of‐GFP‐quantified‐and‐segmented‐cells. 100 randomly sampled cells from both the WT and Δ*emc3* strains were used for the quantitation of signal intensity. Quantified intensities were then compared and analysed using two‐tailed unpaired *t*‐tests and the *P*‐values are written in the appropriate figure legends.

#### Diploid strain generation

The BirA‐Emc6 strain was grown on YPD supplemented with NAT and AviTagged interactors were grown on SD_MSG_ without histidine supplemented with HYG at 30°C overnight. Both strains were velveted onto a YPD plate and grown overnight at RT. The mated strains were then velveted onto SD_MSG_ plates without histidine supplemented with NAT and HYG and grown overnight at 30°C. This step was repeated once more to select diploid strains containing the combination of desired traits.

#### BirA/AviTag interaction assay

Diploid strains were grown overnight at 30°C in SD_MSG_ liquid media without histidine supplemented with NAT, HYG and auxin (1 mM, Sigma). Strains were then back‐diluted to 0.2 OD_600_ and incubated for 4 h at 30°C in SD_MSG_ liquid media without histidine supplemented with NAT, HYG, auxin and biotin (100 nM, Sigma). Cells were collected upon reaching 0.5 OD_600_ by centrifugation at 3,000 *g* for 3 min, washed once in double distilled water (DDW) and then processed for Western blotting (see above).

#### Yeast library generation

SWAT library generation was performed as described (Weill *et al*, 2018). Briefly, a RoToR array pinning robot (Singer Instruments) was used to mate the parental N′ tag GFP SWAT library with the required donor strain (Dataset EV2) and carry out the mating, sporulation and selection protocol to generate a haploid library selected for all the desired features (Tong & Boone, 2007). Growth of the library on YPGalactose (2% peptone, 1% yeast extract, 2% galactose) was used to induce SceI‐mediated tag swapping, and subsequent growth on SD containing 5‐fluoroorotic acid (5‐FOA, Formedium) at 1 g/l, and required metabolic and antibiotic selections was used to select for strains, which had successfully undergone the SWAT process. Information on library genotypes, mating types, SWAp‐Tag efficiency, percentage survival and other quality control checks can be found in Dataset EV5 along with the lists of tagged ORFs for each library.

## Author contributions

**Emma J Fenech:** Conceptualization; data curation; formal analysis; validation; investigation; visualization; methodology; writing – original draft; writing – review and editing. **Nir Cohen:** Conceptualization; data curation; formal analysis; validation; investigation; visualization; methodology; writing – review and editing. **Meital Kupervaser:** Data curation; formal analysis; writing – review and editing. **Zohar Gazi:** Formal analysis; visualization. **Maya Schuldiner:** Conceptualization; resources; supervision; funding acquisition; investigation; writing – original draft; project administration; writing – review and editing.

In addition to the CRediT author contributions listed above, the contributions in detail are:

EJF and NC designed, performed and analysed the experiments. MK ran LC–MS/MS samples and processed the LC–MS/MS data. ZG quantified microscopy data. EJF and MS wrote the manuscript, which all authors read and provided feedback on. MS supervised the work and secured funding.

## Disclosure and competing interests statement

The authors declare that they have no conflict of interest. MS is an editorial advisory board member. This has no bearing on the editorial consideration of this article for publication.

## Supporting information



Expanded View Figures PDF

Dataset EV1

Dataset EV2

Dataset EV3

Dataset EV4

Dataset EV5

PDF+

## Data Availability

Protein interaction IP/AP‐MS data: PRIDE, PXD033348 (http://www.ebi.ac.uk/pride/archive/projects/PXD033348).
