# Supplementary material for: A toolbox for systematic discovery of stable and transient protein interactors in baker's yeast
Source: Mol Syst Biol. 2023 Jan 18;19(2):e11084. doi: 10.15252/msb.202211084 (PMC9912024; doi:10.15252/msb.202211084)
Supplement: Supplementary file 1 — Expanded View Figures PDF [file MSB-19-e11084-s008.pdf]

Expanded View Figures

Figure EV1. Optimising the conditions used for ABOLISH.

A Schematic of the original growth conditions used to test Bpl1-AID*-9myc degradation and endogenous biotinylation reduction.
B Anti-Myc and streptavidin blots of cells expressing Bpl1-AID*-9myc and OsTIR1 grown overnight and back-diluted for ~ 4 h in regular synthetic (SD) or reduced biotin (RB) media. To the cells grown in RB media, auxin was either omitted (−) or added 15 min, 30 min, 1 h or 2 h prior to harvesting. Similarly, biotin was either omitted (−) or added 5 min prior to harvesting. Band intensities for the most prominently endogenously biotinylated proteins in lanes 1, 2, 3 and 7 were quantified using Fiji software.
C Anti-HA blots confirming the expression of BioID2/TurboID-HA-tagged Emc6 and Sbh1. An anti-Myc blot was included for the strains containing the ABOLISH system.
D Western blot analysis of cells expressing either TurboID-HA-Emc6 or TurboID-HA-Sbh1 together with the ABOLISH system. Cells were grown in SD or RB media as in (B). Auxin and biotin were either omitted (−) or added (+) 1.5 h and 30 min, respectively, before harvesting.
E Western blot analysis of cells expressing TurboID-HA-Emc6 and the ABOLISH system. Cells were grown in several different conditions: (i) overnight and back-diluted in rich media (YPD) containing auxin; (ii) overnight and back-diluted in regular SD containing auxin; (iii) overnight and back-diluted in RB media with auxin and biotin either omitted (−), added for 1.5 h and 30 min, respectively, before harvesting (+), or added for the entire duration of the back-dilution (++); (iv) overnight and back-diluted in regular SD with auxin and biotin treatments as in (iii). Sec61 was used as an untagged ER membrane protein control.

Data information: For panels B–E, H3 (histone H3) or Actin was used as loading controls. Back-dilution times were all ~ 4 h.

 © 2023 The Authors

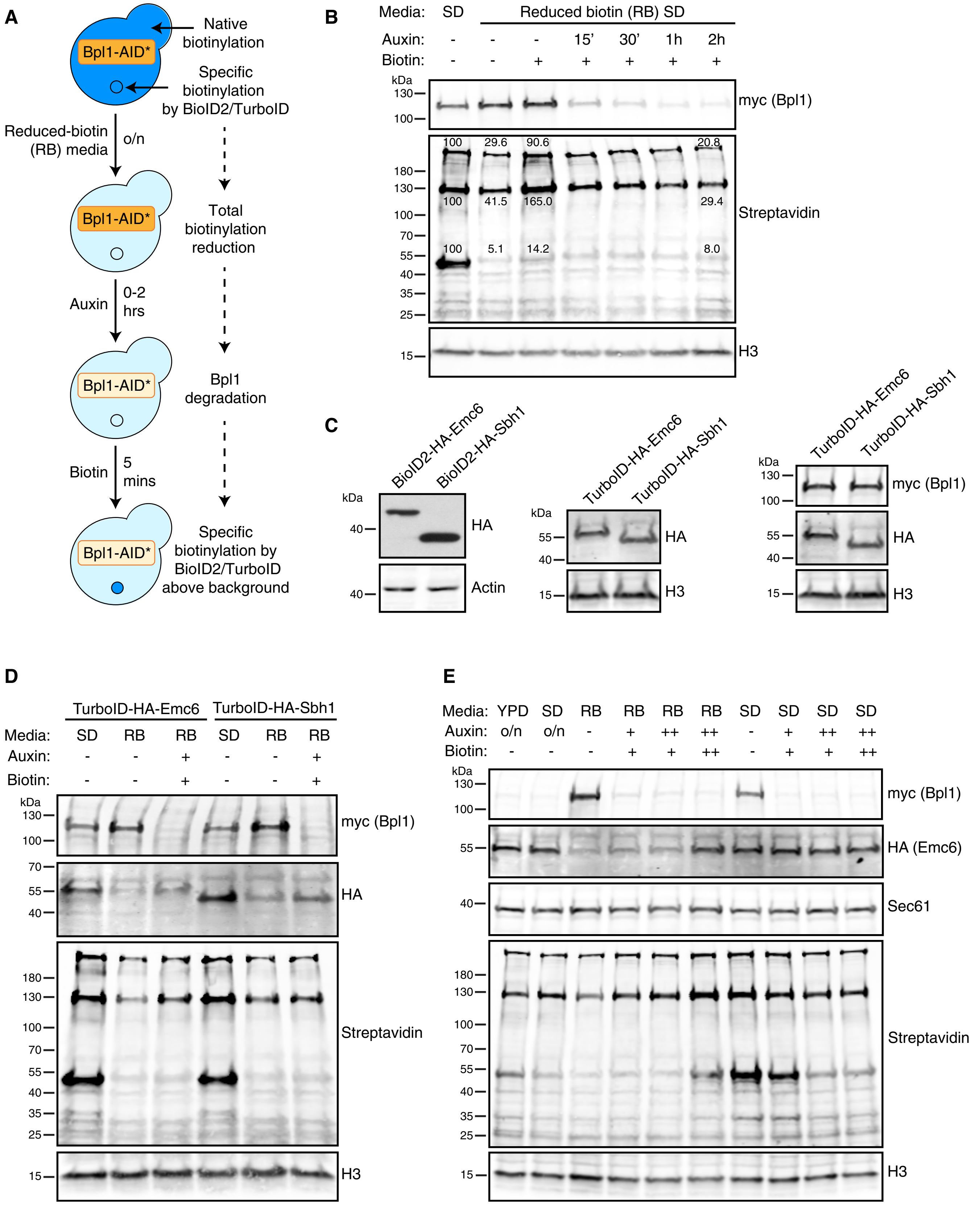
A
Bpl1-AID*
Native biotinylation
Specific biotinylation by BioID2/TurboID
Reduced-biotin (RB) media
o/n
Total biotinylation reduction
Auxin
0-2 hrs
Bpl1 degradation
Biotin
5 mins
Specific biotinylation by BioID2/TurboID above background
B
Media: SD Reduced biotin (RB) SD
Auxin: - - - 15' 30' 1h 2h
Biotin: - - + + + + +
kDa
130
100
myc (Bpl1)
100 29.6 90.6 20.8
100 41.5 165.0 29.4
100 5.1 14.2 8.0
180
130
100
70
55
40
35
25
Streptavidin
15
H3
C
BioID2-HA-Emc6
BioID2-HA-Sbh1
kDa
40
HA
40
Actin
TurboID-HA-Emc6
TurboID-HA-Sbh1
kDa
55
40
HA
15
H3
TurboID-HA-Emc6
TurboID-HA-Sbh1
kDa
130
100
myc (Bpl1)
55
40
HA
15
H3
D
TurboID-HA-Emc6 TurboID-HA-Sbh1
Media: SD RB RB SD RB RB
Auxin: - - + - - +
Biotin: - - + - - +
kDa
130
100
myc (Bpl1)
70
55
40
HA
180
130
100
70
55
40
35
25
Streptavidin
15
H3
E
Media: YPD SD RB RB RB RB SD SD SD SD
Auxin: o/n o/n - + ++ ++ - + ++ ++
Biotin: - - - + + ++ - + + ++
kDa
130
100
myc (Bpl1)
55
HA (Emc6)
40
Sec61
180
130
100
70
55
40
35
25
Streptavidin
15
H3

Figure EV1.

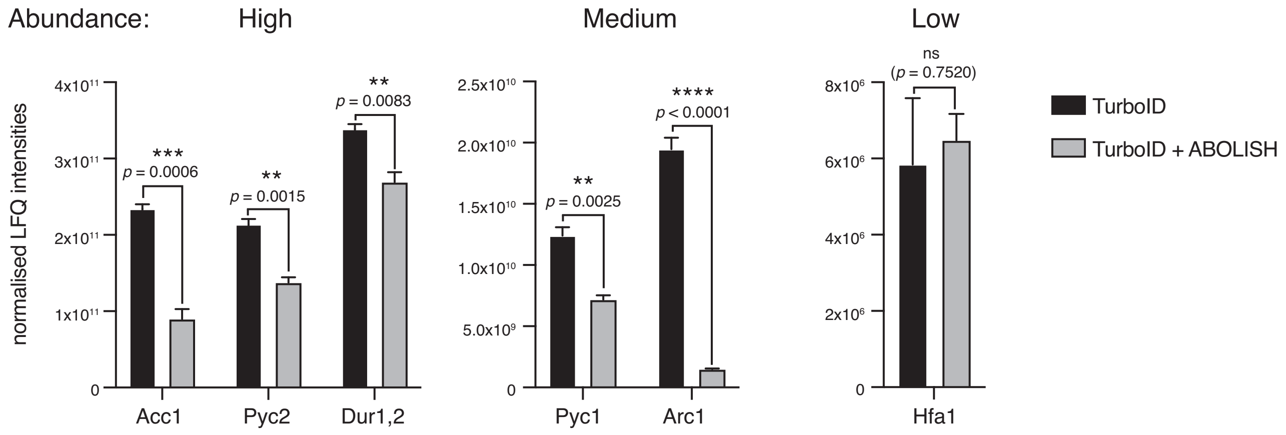

Figure EV2. Quantitation of ABOLISH in LC–MS/MS.

Bar graphs showing the normalised label-free quantification (LFQ) intensities of endogenously biotinylated proteins, with and without ABOLISH, as measured by LC–MS/MS from biological triplicates. Proteins are grouped into either high, medium or low abundance to enable clear visualisation of the differences. Shown are the standard error of the mean (SEM) and *P*-values from two-tailed unpaired *t*-tests demonstrating significance or not (ns).

© 2023 The Authors

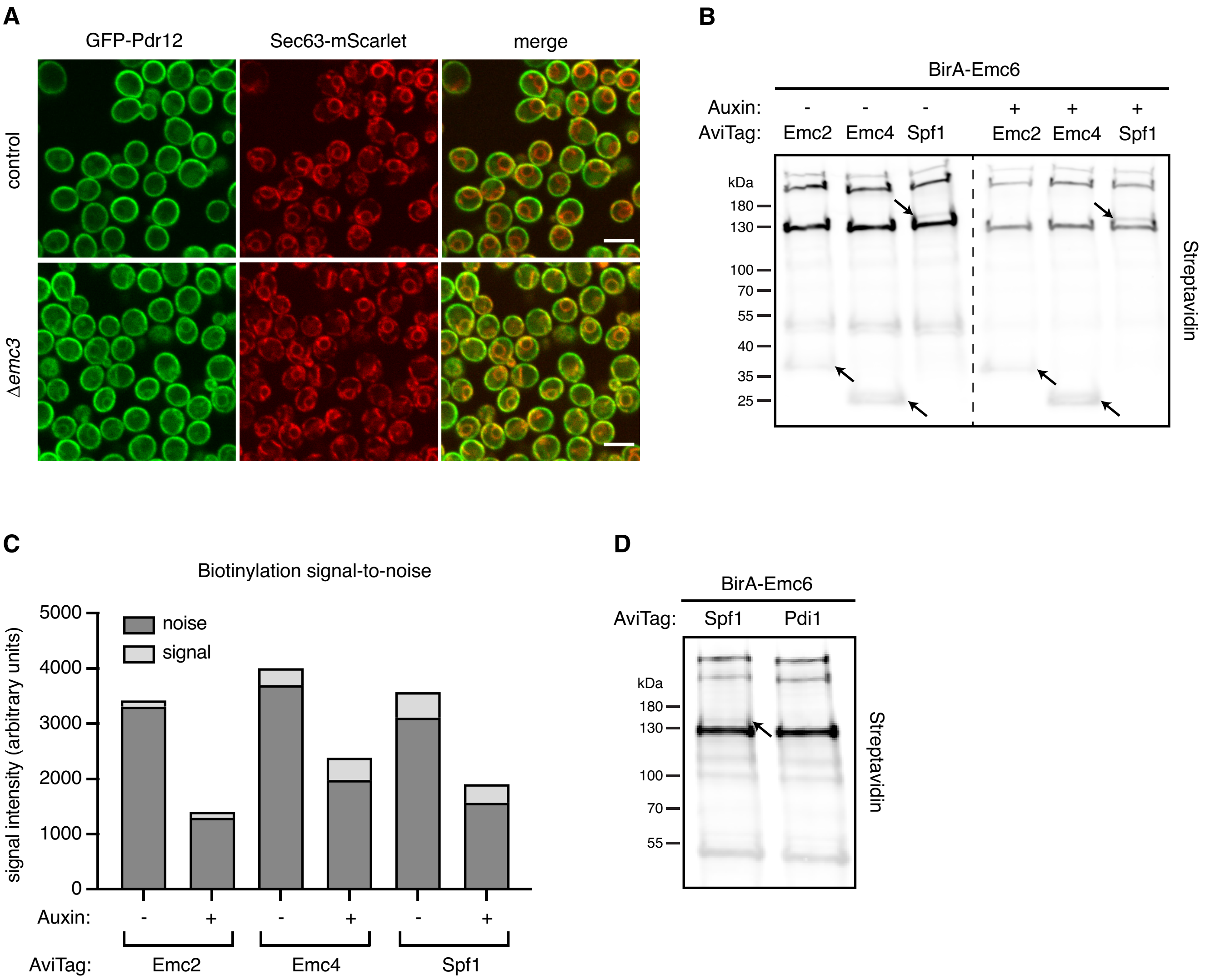

Figure EV3. Contribution of the ABOLISH system to noise reduction in BirA-AviTag blotting.

A Fluorescence microscopy images of control and Δ*emc3* strains containing Pdr12 N′ tagged with GFP and Sec63 C′ tagged with mScarlet. Sec63 localisation was unchanged upon loss of Emc3 and no significant difference was found in signal intensity ($P = 0.1156$). Scale bar = 5 μm.

B Streptavidin blot of diploid strains expressing either AviTag-Emc2, -Emc4, or -Spf1 together with BirA-Emc6, grown in media with or without auxin. 15 μg of whole-cell lysate from each sample was loaded onto the gel. The arrows indicate the bands corresponding to the molecular weight of each of the AviTagged proteins.

C Quantitation of the streptavidin signal from endogenously biotinylated proteins (noise) and biotinylated AviTagged proteins (signal) show a reduction in the background noise by ~half when ABOLISH is activated.

D Streptavidin blot of diploid strains expressing either AviTag-Spf1 or -Pdi1 together with BirA-Emc6, grown in media containing auxin. The arrow indicates the band corresponding to the molecular weight of the AviTagged protein.

Figure EV4. Quality control checks on biotinylation toolkit libraries.

A–C Western blot analysis for selected strains from the BioID2-HA (A) TurboID-HA (B) and TurboID-HA/ABOLISH (C) libraries. For the BioID2-HA library, Phm6, Din7, Skt5, Rcr1 and Mig2 all have a low endogenous expression (relative intensity values of ≤ 27, Weill *et al*, 2018), which likely explains why they were not readily detectable. ‘Control (28)’ refers to BioID2-HA not tagged to any protein, and ‘WT’ denotes lysate ran from the BY4741 laboratory strain to highlight nonspecific bands; the most prominent of which are marked with an asterisk. For all panels, H3 (histone H3) is used as a loading control. The expected molecular weight in kDa for each tested protein including their tag is written in parentheses after the protein name.

▶

© 2023 The Authors

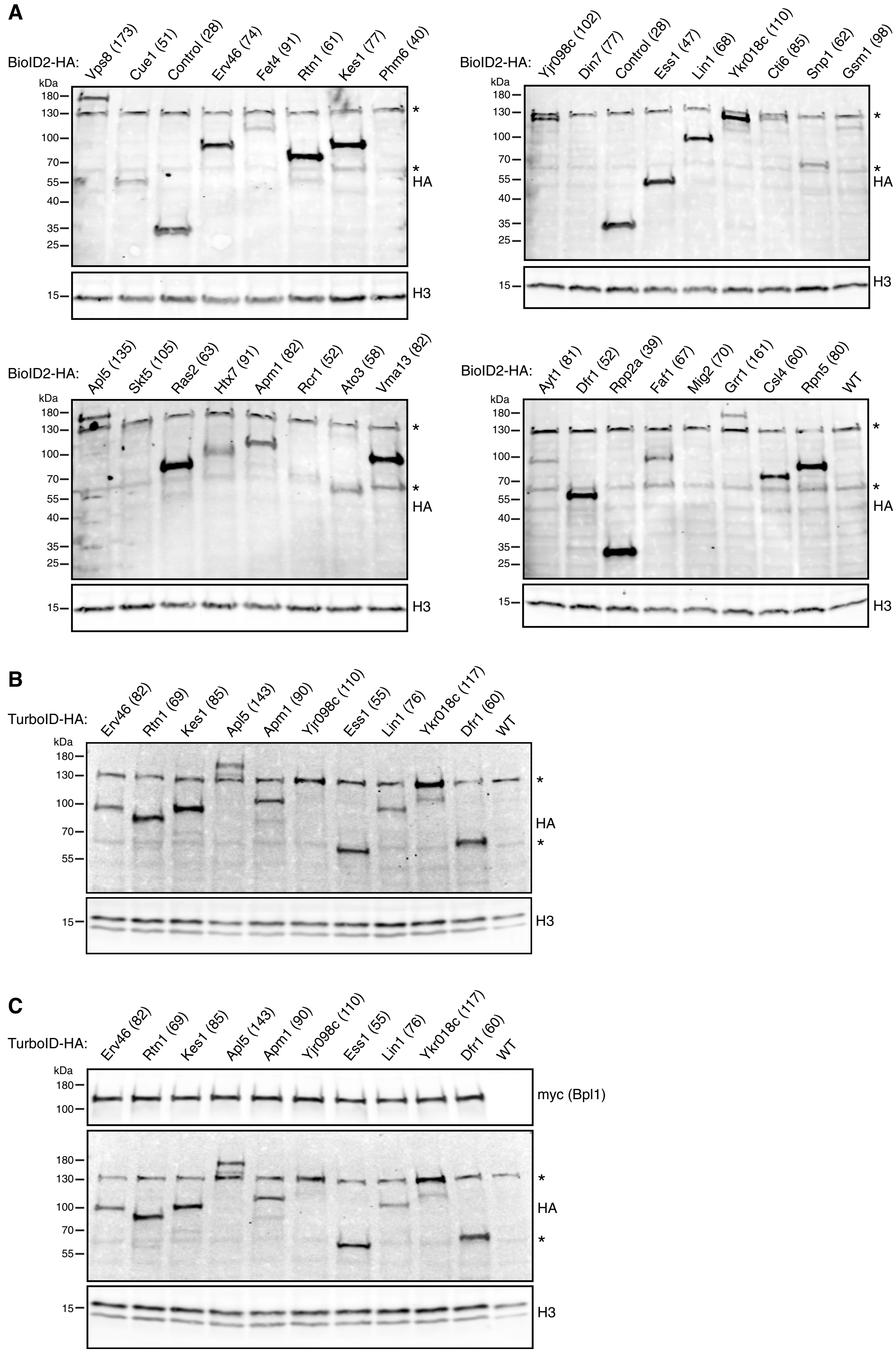

Figure EV4.

 © 2023 The Authors
